# Cold Exposure Induces Depot-Specific Alterations in Fatty Acid Composition and Transcriptional Profile in Adipose Tissues of Pigs

**DOI:** 10.3389/fendo.2022.827523

**Published:** 2022-02-23

**Authors:** Yanbing Zhou, Ziye Xu, Liyi Wang, Defeng Ling, Qiuyun Nong, Jintang Xie, Xiaodong Zhu, Tizhong Shan

**Affiliations:** ^1^ College of Animal Sciences, Zhejiang University, Hangzhou, China; ^2^ Key Laboratory of Molecular Animal Nutrition (Zhejiang University), Ministry of Education, Hangzhou, China; ^3^ Key Laboratory of Animal Feed and Nutrition of Zhejiang Province, College of Animal Sciences, Zhejiang University, Hangzhou, China; ^4^ Shandong Chunteng Food Co. Ltd., Zaozhuang, China

**Keywords:** cold exposure, pigs, adipose tissue, fatty acid metabolism, transcriptome, metabolome

## Abstract

Cold exposure promotes fat oxidation and modulates the energy metabolism in adipose tissue through multiple mechanisms. However, it is still unclear about heat-generating capacity and lipid mobilization of different fat depots without functional mitochondrial uncoupling protein 1 (UCP1). In this study, we kept finishing pigs (lack a functional UCP1 gene) under cold (5-7°C) or room temperature (22-25°C) and determined the effects of overnight cold exposure on fatty acid composition and transcriptional profiles of subcutaneous adipose tissue (SAT) and visceral adipose tissue (VAT). And the plasma metabolomes of porcine was also studied by LC-MS-based untargeted metabolomics. We found that the saturated fatty acids (SFAs) content was decreased in SAT upon cold exposure. While in VAT, the relative content of lauric acid (C12:0), myristic acid (C14:0) and lignoceric acid (C24:0) were decreased without affecting total SFA content. RNA-seq results showed SAT possess active organic acid metabolism and energy mobilization upon cold exposure. Compared with SAT, cold-induced transcriptional changes were far less broad in VAT, and the differentially expressed genes (DEGs) were mainly enriched in fat cell differentiation and cell proliferation. Moreover, we found that the contents of organic acids like creatine, acamprosate, DL-3-phenyllactic acid and taurine were increased in plasma upon overnight cold treatment, suggesting that cold exposure induced lipid and fatty acid metabolism in white adipose tissue (WAT) might be regulated by functions of organic acids. These results provide new insights into the effects of short-term cold exposure on lipid metabolism in adipose tissues without functional UCP1.

## Introduction

The global epidemic of metabolic syndrome has become the major health hazard of the modern world (1). The syndrome is mostly driven by excess energy intake and concomitant obesity (2). To control the ongoing obesity epidemic, both active lifestyle and new treatment approaches to induce weight loss are required. Homeothermic animals exposed to low ambient temperatures activate adaptive thermogenesis, including shivering and facultative thermogenesis, to increase mobilization of energy to maintain body temperature. The most important heat source from cold exposure is facultative thermogenesis, which increases metabolic reactions by activating BAT function and browning of WAT through the activation of β adrenergic signaling in both humans and mice (3, 4). As a process of heat generation, cold exposure enhanced the cycle using and removing free fatty acid (FFA) from blood to disrupt energy accumulation in adipose tissues (3). In the past decades, targeting the activity of BAT and browning of WAT to increase energy expenditure is a promising strategy to combat obesity.

Most studies on cold induced BAT activation have been conducted in rodents and few human trials exist to illuminate functions of BAT upon cold exposure (5). And the volume of active BAT in adult humans is highly variable with each individual, which is associated with gender, age, and body-mass index (BMI) (5–7). Therefore, the contribution of BAT to overall energy metabolism in individuals becomes unclear. In recent years, studies have shown that inactivation of uncoupling protein 1 (UCP1), the central element of heat production in BAT, did not potentiate diet-induced obesity (8), and also not require in long-term cold adaptation (9). Recent studies have also confirmed the existence of multiple thermogenic mechanisms, which are based on adenosine triphosphate (ATP) sinks and fatty acid-mediated UCP1-independent leak pathways driven by the adenosine diphosphate (ADP)/ATP carrier (8, 10, 11). On account of the complicated in UCP1-dependent heat generation, further studies are carried out in UCP1-KO models to accurately measure the difference between UCP1-independent and UCP1-dependent thermogenesis to better identify pharmacological products that mimic the cold-induced heat production on adipose tissues. Pig (Sus scrofa domesticus) is a species with the absence of a functional UCP1 (12). Lack of functional UCP1 makes modern pigs cold sensitive (13). Pig is a human-sized omnivorous animal and closely related to humans in terms of anatomy, genetics and physiology. As a major species for livestock production for thousands of years, pig also extended its use as a preferred model species for analyses of a wide range of human physiological functions and diseases (14–16). Since recent studies have focused on exploring cold-induced UCP1-independent thermogenesis mechanism and improving thermogenic capacity of piglets (17). Little attention has been paid to the response of metabolized processes of finishing pigs under cold stress. And the heterogeneity between subcutaneous adipose tissue (SAT) and visceral adipose tissue (VAT) in pigs upon cold exposure has not been described. Accordingly, cold-induced physiological changes in porcine SAT and VAT have referential value in drawing the mechanism of UCP1-independent heat generating by dissipating stored chemical energy on human adipose tissues, which may contribute to the finding of new therapies for epidemic obesity treatment.

In this study, we kept finishing pigs upon cold or room temperature and investigated the effects of overnight cold exposure on triglyceride (TG) content, enzyme activity, fatty acid composition, gene expression profiles in white adipose tissues (SAT and VAT) and metabolic changes in plasma. We revealed that cold-induced alterations in fatty acid composition and transcriptome profiles are depot-specific in porcine adipose tissues. Moreover, combined with metabolome analyses, we conclude that porcine SAT might retain part of heat-generating capacity that could be driven by circulating organic acids.

## Materials and Methods

### Animals and Samples

Twelve DLY (Duroc × Landrace × Yorkshire) pigs at slaughter weight (120~125 kg) were used to investigate the cold-induced changes in fatty acid composition, gene expression profiles in adipose tissues and metabolic profiles in blood. The experimental design was performed as previously published methods (18). Briefly, six pigs of one group were housed at cold condition (COLD, 5-7°C), and six in the control group were housed at room temperature condition (RT, 22-25°C) overnight (14 h) without feed but free access to water. The SAT, VAT and blood were sampled immediately after cold exposure and quickly frozen in liquid nitrogen and stored at −80°C immediately for subsequent analysis.

### Backfat Thickness Measurement

Pigs were slaughtered by exsanguination after electric stunning (90~100 V, 0.9-1.0 A, 50 Hz) then hoisted and followed by bleeding, dehairing and eviscerating in a commercial abattoir. The whole process was completed about 20 min post mortem. Backfat thickness was determined by average scores of first- and last-rib, and last-lumbar of the right carcass sides.

### Hematoxylin-Eosin Staining

Hematoxylin-eosin (H&E) staining of SAT and VAT samples from RT and COLD pigs were performed as previously published methods (19). Adipose tissues of pigs were fixed at room temperature in 10% formalin for 24 h. Next, the tissues were embedded into paraffin, blocked, and cut at 10 μm for staining. The adipose tissue sections were deparaffinized, rehydrated, and stained with hematoxylin for 15 min. Then sections were rinsed in running tap water and stained with eosin for about 5 min, dehydrated, mounted, captured.

### Triacylglycerol (TG) and Total Cholesterol (TCHO) Measurement

The left-half carcasses of SAT and VAT samples were used for lipids content measurement. The contents of TG and TCHO were measured as previously published methods (18).

### Enzyme Activities Analysis

For oxidative stress indices measurement, sample were prepared as previously published methods (18). The activities of total antioxidant capacity (T-AOC), catalase (CAT), lactate dehydrogenase (LDH) and peroxidase (POD) were measured by using commercial kits (T-AOC, A015-2; CAT, A007-1; LDH, A020-2-2; POD, A084-1) bought from Nanjing Jian Cheng Institute of Bioengineering (Nanjing, Jiangsu, China).

### Fatty Acid Profiles Analysis

Free fatty acid mixtures were obtained from SAT and VAT after extracted and hydrolyzed in 2 mL KOH-methanol. Fatty acid profiles of SAT and VAT from RT and COLD were analyzed as previously published methods (18).

### RNA Isolation, Library Construction, RNA-Seq and Quantitative Real-Time PCR

RNA extraction, library construction, RNA-seq analysis and quantitative real-time PCR (qPCR) of SAT and VAT samples from RT and COLD pigs were performed as previously published methods (19). Primers used for qPCR are shown in **Supplementary Table 3**.

### Untargeted Metabolomics Relative-Quantitative Analysis

Twelve plasma samples (6 RT and 6 COLD) were used for metabolomics analysis. The plasma samples were thawed and mixed with cold methanol/acetonitrile/H_2_O (2:2:1, v/v/v, 1mL) and sonicated for 30 min (twice) then centrifuged at 14000 g for 20 min (operation at 4° C). The supernatant was dried in a vacuum centrifuge.

For LC-MS analysis, the samples were re-dissolved in 100 μl acetonitrile/water (1:1, v/v) solvent, then put in an automatic sampler at 4°C during the experiment. Analyses were performed using an UHPLC (1290 Infinity LC, Agilent Technologies) coupled to a quadrupole time-of-flight (AB Sciex Triple TOF 6600) as published (20) with modifications in Shanghai Applied Protein Technology Co., Ltd.

The raw MS data were converted to MzXML files by Proteo-Wizard MS Convert then imported into freely available XCMS software. After peak alignment and retention time correction, peak area was extracted. Only the variables having more than 50% of the nonzero measurement values were kept. Compound identification of metabolites with an in-house database established with available authentic standards. After normalized, the processed data were imported into SIMCA-P (version 16.1, Umetrics, Umea, Sweden), and subjected to Pareto-scaled principal component analysis (PCA) and orthogonal partial least-squares discriminant analysis (OPLS-DA). The robustness of the model was evaluated by 7-fold cross-validation and response permutation testing. The variable importance in the projection (VIP) value of each variable in the OPLS-DA model was calculated. Metabolites with the VIP value >1 was further applied to Student’s t-test, the P values less than 0.05 were considered as statistically significant.

### Pathway Enrichment Assay

Gene Ontology (GO) and Kyoto Encyclopedia of Genes and Genomes (KEGG) analyses were performed to identify differentially expressed genes (DEGs) which were significantly enriched in GO terms or metabolic pathways as previously published methods (19). GO terms and KEGG pathways with false discovery rates P < 0.05 (the p-values were adjusted using the Benjamini & Hochberg method) were considered as significantly altered. Enriched terms and pathways were visualized by centupled and metaplot function. KEGG pathway enrichment analyses was performed to explore the impact of differentially expressed metabolites. Analyses were applied based on the Fisher’ exact test, considering the whole metabolites of each pathway as background dataset. And only pathways with p-values under a threshold of 0.05 were considered as significant.

### Data Analysis

Data on TG contents, enzyme activities, fatty acid composition, TPM level and relative mRNA expression level were presented as the mean ± SEM. Data were analyzed by unpaired two-tailed Student’s t-tests. Data visualization and statistical analyses were performed using the GraphPad Prism 9.0.0 software package (Monrovia, CA, USA) and R software (version 4.0.5). Differences between groups were considered statistically significant at *P* < 0.05.

## Results

### Cold Exposure Induced Depot-Specific Response on Lipid Contents and Oxidative Balance in Porcine Adipose Tissues

**Figure 1:**
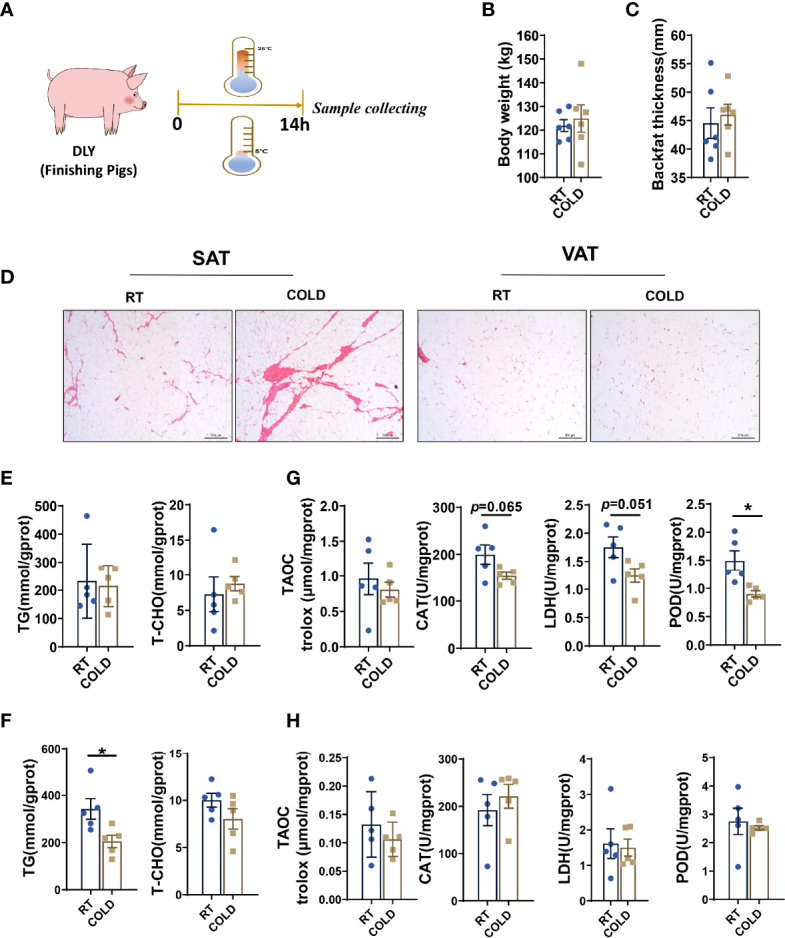
Effect of cold exposure on carcass characteristics, TG and TCHO contents and various enzymes activities. **(A)** Scheme of the experimental process. **(B)** Body weight of pigs under RT (room temperature) or COLD (cold exposure) treatment. n=6 for each group. **(C)** Backfat thickness. **(D)** H & E staining of SAT and VAT sections from RT and COLD pigs. Scale bars, 300 μm (n=3). **(E)** TG and TCHO contents in SAT. **(F)** TG and TCHO contents in VAT. **(G)** Changes of antioxidant enzymes activities in SAT. **(H)** Changes of enzymes activities in VAT. Data are presented as mean ± SEM (n = 5). *P < 0.05, two-tailed Student’s t-test.

Twelve DLY pigs at slaughter weight were used to investigate the cold-induced changes in different adipose tissues (**Figure 1A**). The body weights (BW) of the pigs were similar (**Figure 1B**). There were no significant differences in backfat thickness between COLD and RT pigs (**Figure 1C**). Notably, an obvious increase in angiogenesis of SAT was found upon cold exposure (**Figure 1D**). And the TG and TCHO contents in porcine SAT (**Figure 1E**) and VAT were measured (**Figure 1F**). We found that TG content was not affected after overnight cold exposure in SAT while TG level was significantly decreased after cold-treated in VAT (**Figure 1F**). However, inconformity with the TG content, an obvious decrease of adipocyte cell size was not observed in VAT after cold treatment (**Figure 1D**). To explore the changes of oxidative and anti-oxidative balance in SAT and VAT upon cold exposure, we measured enzyme activities associated with antioxidation, oxidative stress and lipid oxidative (**Figures 1G, H**). TAOC, which is responsible for antioxidant capacity, showed no change after cold exposure, in SAT (**Figure 1G**) and VAT (**Figure 1H**). The biomarkers of oxidative stress, CAT and LDH, had a decreasing tendency in SAT (**Figure 1G**) of cold-treated pigs, but not in VAT (**Figure 1H**). Moreover, the activity of POD, which associates with lipid oxidative, was decreased in SAT after cold treatment (**Figure 1G**). These data suggest that overnight cold exposure increased triglyceride breakdown in porcine VAT and decreased the oxidative stress and lipid peroxidation in SAT.

### Alteration in Fatty Acid Profiles of SAT and VAT Upon Cold Exposure

**Figure 2:**
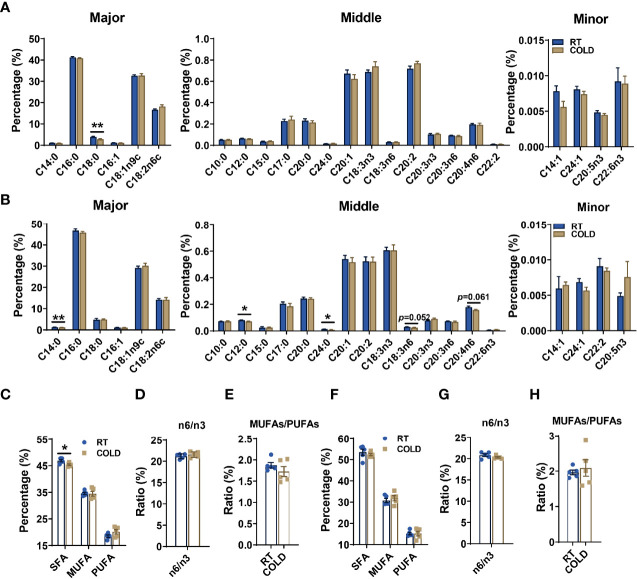
Cold exposure changed the composition and proportions of fatty acids. Fatty acid composition analyses of SAT and VAT isolated from pigs maintained at RT or in COLD for 14 hours. **(A)** The relative concentration of individual fatty acids in SAT from RT and COLD pigs. Fatty acids are divided into major, middle and minor species based on abundance. Fatty acids are sorted by a degree of saturation. **(B)** The relative concentration of individual fatty acids in VAT from RT and COLD pigs. **(C)** The percentages of total SFAs, MUFAs and PUFAs in SAT from RT and COLD pigs. SFAs, saturated fatty acids; MUFAs, monounsaturated fatty acids; PUFAs, polyunsaturated fatty acids containing two or three to six double bonds. **(D)** The ratio of n6-fatty acids: n3-fatty acids (n6: n3) and **(E)** MUFAs: PUFAs in SAT from RT and COLD pigs. **(F)** The percentages of total SFAs, MUFAs and PUFAs in VAT from RT and COLD pigs. **(G)** The ratio of n6-fatty acids: n3-fatty acids (n6: n3) and **(H)** MUFAs: PUFAs in VAT from RT and COLD pigs. n = 5. Error bars represent S.E.M. *P < 0.05, **P < 0.01, two-tailed Student’s t-test.

Next, we analyzed the fatty acid composition in adipose tissue of COLD and RT pigs. Absolute proportions showed that overnight cold exposure did not cause an extensive change of fatty acid contents in porcine SAT (**Supplementary Table 1**) and VAT (**Supplementary Table 2**). Notably, cold-treatment significantly reduced relative stearic acid (C18:0) in SAT (**Figure 2A**). In VAT, cold exposure induced reduction of the relative contents of following fatty acids: lauric acid (C12:0), myristic acid (C14:0) and lignoceric acid (C24:0). γ-linolenic(C18:3n6) and arachidonic acid (C20:4n6), which are known for threatening human health, had a decreasing tendency (**Figure 2B**). Besides, we calculated the percentages of SFAs, MUFAs, PUFAs and the ratio of MUFAs: PUFAs n6-fatty acids: n3-fatty acids (n6: n3) in SAT and VAT (**Figures 2C–H**). In line with decreasing stearic acid content, the relative percentage of SFAs was significantly decreased in COLD SAT (**Figure 2C**). Although it performed more extensive influence on individual fatty acids content than SAT, cold exposure did not affect fatty acid profiles in VAT (**Figure 2F**). These results suggest that cold exposure reduced total SFA content in SAT, while it tended to affect individual fatty acid content in VAT.

### Cold Exposure Changed the Transcriptome Profiles of SAT in Pigs

**Figure 3:**
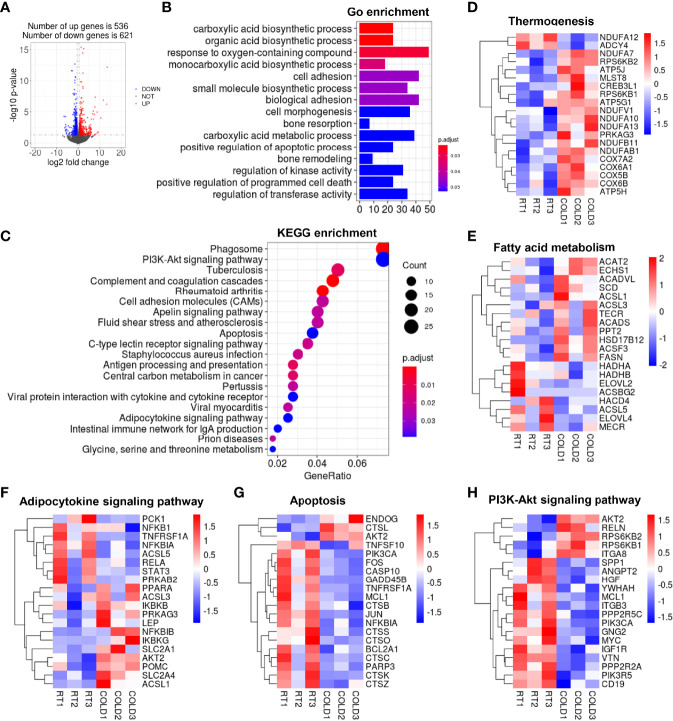
The changes of transcriptome profile in porcine SAT after cold exposure. **(A)** Volcano plot of differently expressed genes (DEGs). Log2 fold changes in exons of RNA-seq gene bodies in COLD versus RT pigs and the corresponding significance values displayed as log10 (P value). In total, 536 and 621 genes were identified that had induced (red) or repressed (blue) expression levels by cold exposure, gray denotes genes with no significant changes. **(B)** Gene Ontology (GO) enrichment analysis. **(C)** Functional enrichment analyses using Kyoto Encyclopedia of Genes and Genomes (KEGG) pathways. **(D, E)** Heatmaps of the TPM expression values of selected thermogenesis, fatty acid metabolism regulated genes from the RNA-seq dataset. **(F–H)** KEGG results of the cold-induced enrichment of genes involved in adipocytokine signaling pathway **(F)**, apoptosis **(G)**, PI3K-Akt signaling pathway **(H)**.

To further investigate the alteration of transcription profiles following cold exposure, fat tissues from different depots, including subcutaneous and visceral depots, were collected from cold-treated and control pigs and subjected to RNA-seq to map the transcriptional changes. Volcano plots exhibited a broad overview alteration in gene expression between these two groups. 1157 DEGs were identified in the COLD and RT group using the filter criteria of Log2 (fold change) > 1 and p-value < 0.05, of which 536 were increased, and 621were decreased in SAT after cold-treated (**Figure 3A**). GO enrichment analysis (21) showed that overnight cold exposure affected biological processes in SAT, including carboxylic acid metabolic processes, organic acid biosynthetic process, response to oxygen-containing compound and monocarboxylic acid biosynthetic process (**Figure 3B**). The cneplot showed that a huge amount of DEGs related to the organic acid biosynthetic process was regulated, including early growth response 1 (*EGR1*), solute carrier 2A4 (*SLC2A4*), solute carrier 2A8 (*SLC2A8*), insulin-like growth factor 1 receptor (*IGF1R*), neutralized E3 ubiquitin protein ligase 1 (*NEURL1*), regulator of G-protein signaling 10 (RGS10) (**Supplementary Figure 1A**). Functional enrichment analyses using the KEGG pathways (22) enrichment analysis revealed that DEGs significant enrichment in PI3K-Akt signaling pathway, adipocytokine signaling pathway, apoptosis (**Figure 3C**) and immune response-related pathways centered on the phagosome (**Supplementary Figure 1B**). To further explore the processes of heat generation and fatty acid oxidation in adipocytes under cold exposure, we analyzed the genes related to thermogenesis and fatty acid metabolism. The expression of genes encodes NADH-ubiquinone oxidoreductase (*NDUs*), cytochrome C oxidase (*COXs*) and *ATPs* were up-regulated in SAT of pigs upon cold exposure (**Figure 3D**). They are essential for the function of mitochondrial oxidative phosphorylation system. Expression levels of genes involved in fatty acid metabolism associated pathways including fatty acid elongation (*ELOVL2*, *ELOVL4*, *HACD4*), mitochondrial fatty acid synthesis (MECR), long chain fatty acyl-CoA β-oxidation (HADHA, HADHB), fatty acid β-oxidation (ACSL1, ACSL3, ACSF3, ACADVL, ECHS1) and *de novo* fatty acid synthesis (SCD, FASN, TCER) were given in **Figure 3E**. Besides, cold treatment influenced glucose uptake and gluconeogenesis, the expression of glucose transporters (SLC2A1, SLC2A4) was up-regulated, the expression of phosphoenolpyruvate carboxykinase (PCK) was down-regulated (**Figure 3F**). Moreover, the expression of apoptosis-related genes (GADD45b, CASP10, BCL2a) and inflammation-linked genes (CTSB, CTSC, CTSZ, etc.) were down-regulated after cold exposure (**Figure 3G**). In addition, we found that mTOR1-related genes ribosomal S6 kinase 1(S6K1) and ribosomal S6 kinase 2 (S6K2) were up-regulated (**Figure 3H**), the activation of mTOR signaling in adipose tissue could promote mitochondrial biogenesis and browning reportedly (23). These results suggest that overnight cold exposure enhanced the carboxylic acid biosynthetic process and oxidative phosphorylation related genes but decreased the immune response related genes in SAT.

### Cold Exposure Regulated the Fatty Acid Metabolism and Cellular Processes Related Pathways in Porcine VAT

**Figure 4:**
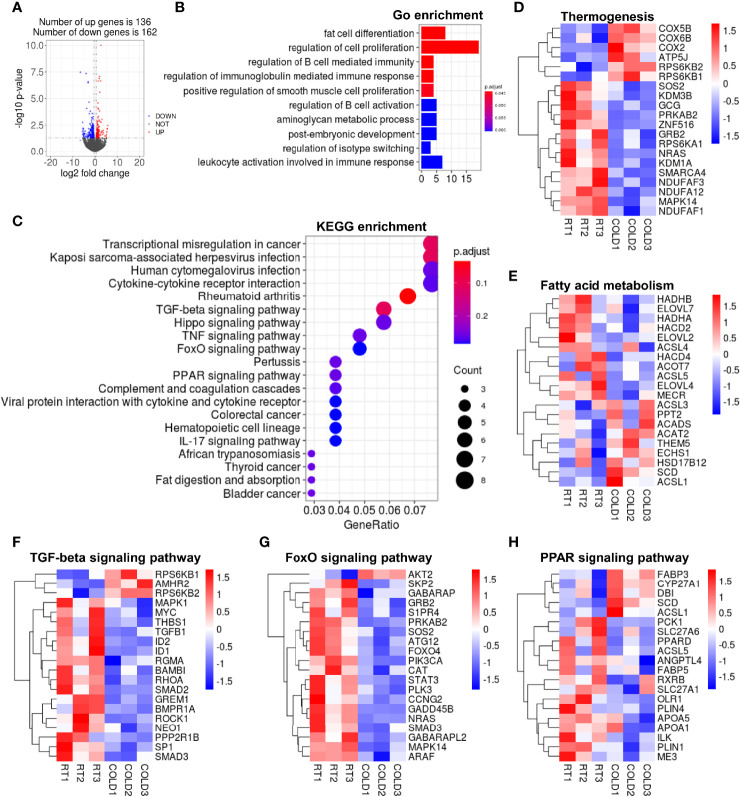
Cold exposure changed the transcriptome profile of VAT in pigs. **(A)** Volcano plot of differently expressed genes (DEGs) expression patterns were illustrated. Red denotes upregulated genes in COLD VAT; blue denotes downregulated genes in COLD VAT; gray denotes genes with no significant changes. **(B)** Gene Ontology (GO) enrichment analysis. **(C)** Functional enrichment analyses using Kyoto Encyclopedia of Genes and Genomes (KEGG) pathways. **(D, E)** Heatmaps of the TPM expression values of selected thermogenesis, fatty acid metabolism regulated genes from the RNA-seq dataset. **(F–H)** KEGG results of the cold-induced enrichment of genes involved in TGF-beta signaling pathway **(F)**, FoxO signaling pathway **(G)**, PPAR signaling pathway **(H)**.

In VAT, 289 DEGs were identified in the COLD and RT group using the filter criteria of | Log2 (fold change) > 1 and p-value < 0.05, of which 136 were increased and 162 were decreased after cold-treated (**Figure 4A**). GO enrichment analysis showed that overnight cold exposure affected the expression of genes related to the biological processes including fat cell differentiation and regulation of cell proliferation in VAT (**Figure 4B**). The transcription of cell proliferation relative genes including vascular endothelial growth factor A (*VEGFA*), heparin-binding epidermal growth factor (EGF)-like growth factor (*HBEGF*), fibroblast growth factor 18 (*FGF18*) was increased (**Supplementary Figure 2A**). The KEGG pathways enrichment analysis revealed that DEGs were significantly enriched in TGF-beta signaling pathway, PPAR signaling pathway, FOXO signaling pathway (**Figure 4C**) and immune response related pathways cored in rheumatoid arthritis (**Supplementary Figure 2B**). Transcriptomic analysis yielded that the expression of thermogenesis related genes *COX5B*、*COX6B* and *COX2* was up regulated in VAT after cold exposure. Contrary to *COXs* genes, the expression of *NDUFs* genes was down-regulated upon cold exposure (**Figure 4D**). Heatmap showed that overnight cold exposure has minimal effect on fatty acid metabolism (**Figure 4E**). According to KEGG enrichment analysis, the transcription of genes enriched in TGF-β signaling pathway and FOXO signaling pathway were almost inhibited in VAT after cold-treated (**Figures 4F, G**). In addition, our RNA-seq analysis showed that the expression of the adipose differentiation-related genes, including perilipin1 (*PLIN1*), perilipin4 (*PLIN4*) and lipid transport-related genes apolipoprotein A1 (*APOA1*), apolipoprotein A5 (*APOA5*) was decreased upon cold treatment (**Figure 4H**). Taken together, the transcription of genes enriched in cell differentiation, cell proliferation and immune response was partly enhanced following cold exposure in VAT. Compared to SAT, the role of cold exposure in transcriptome remodeling is more slightly in VAT.

### Short Term Cold Exposure Impaired the Coherence of Heat-Production and Fatty Acid Metabolism in Pig Adipose Tissues

**Figure 5:**
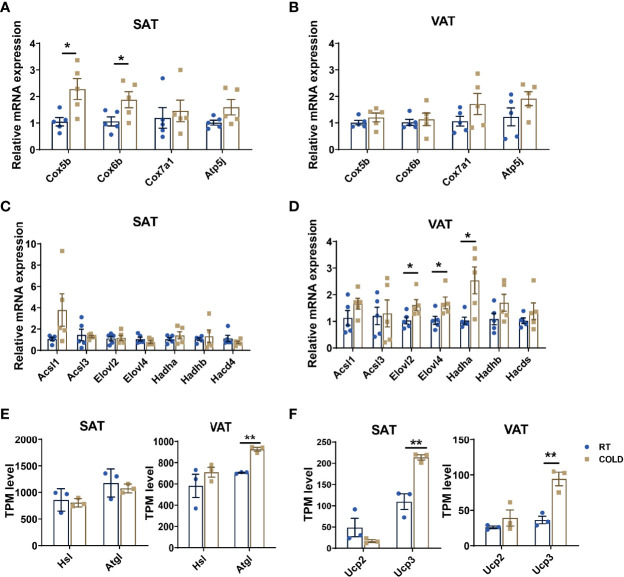
Cold exposure induced alterations in heat production processes. **(A–D)** qPCR validation of the expression of genes related to thermogenesis and fatty acid metabolism in SAT and VAT from RT and cold-treated pigs (n = 5). **(E, F)** Transcripts per million (TPM) expression values of lipolysis markers (*ATGL*, *HSL*) and cold-induced genes (*UCP2*, *UCP3*) of SAT and VAT from cold-treated and RT pigs were shown to estimate relative gene expression abundance (n = 3). Error bars represent SEM. *P < 0.05, **P < 0.01, two-tailed Student’s t-test.

Next, we confirmed the upregulated genes related to mitochondrial oxidative phosphorylation by qPCR, which revealed by heat map before, showed that COX5B and COX6B were upregulated by cold exposure in SAT but not VAT (**Figures 5A, B**). We detected the co-regulated genes between SAT and VAT under cold exposure related to fatty acid metabolism, and we found ELOVL2, ELOVL4 and HADHA were uniquely upregulated in VAT (**Figures 5C, D**). These results indicated that the oxidative phosphorylation of fatty acids was out of step with its anabolism and catabolism in porcine adipose tissue upon cold treated. Hence, we compared the cold-induced transcriptional changes of key genes that contributed to lipolysis and thermogenesis in SAT and VAT. Adipose triglyceride lipase (ATGL) and hormone-sensitive lipase (HSL), which participate in lipolysis constitute, were not significantly altered in COLD SAT (**Figure 5E**). While the expression of ATGL was significantly altered by cold exposure in VAT (**Figure 5E**). Thermogenesis activated by cold exposure depends largely on UCP activity, we found that the mRNA expression level of UCP3 was higher in SAT than VAT upon cold treated (**Figure 5F**). These results suggest that cold-induced transcriptional responses in adipose tissues of pigs is depot-specific.

### Over-Night Cold Exposure Changes the Composition of Plasma Metabolites

**Figure 6:**
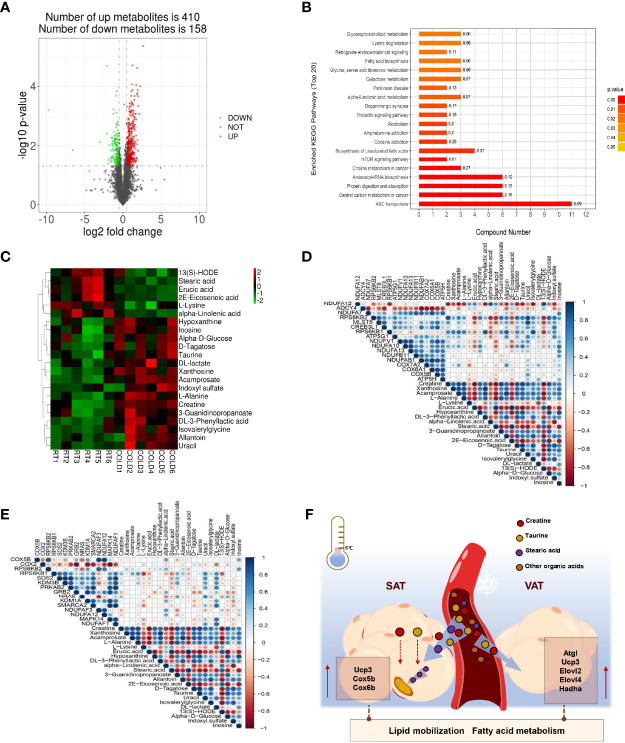
Effect of cold exposure on metabolites of pigs. **(A)** Volcano plot of pairwise comparisons of all detected negative ions in plasma. The threshold (|log2-fold change| > log2 1.5; P value < 0.05) was defined for each significantly changed metabolites. Red denotes upregulated metabolites in COLD plasma; green denotes downregulated metabolites in COLD plasma; gray denotes metabolites with no significant changes. **(B)** KEGG pathway annotation of top 20 differential metabolites. **(C)** A heatmap was drawn to show the differential expressed metabolites. Up-regulated expressed metabolites were shown in red; down-regulated expressed metabolites were shown in green. Metabolites were selected from the metabolomics dataset. Only metabolites with *P* < 0.05 were displayed. **(D)** Heatmap of Pearson correlation coefficients between differential expressed metabolites identified in plasma and thermogenesis relative DEGs in SAT upon cold exposure. Blue denotes positive correlation, Red denotes negative correlation. **(E)** Heatmap of Pearson correlation coefficients between differential expressed metabolites identified in plasma and thermogenesis relative DEGs in VAT upon cold exposure. Blue denotes positive correlation, red denotes negative correlation. **(F)** Working model of the metabolic effects between blood and adipose tissues under cold exposure.

To better determine the effects of blood metabolites on WAT metabolic adaptation to cold exposure, we performed metabolomics analyses on plasma samples collected from RT and COLD pigs. We detected 568 metabolites, including 410 upregulated and 158 downregulated (**Figure 6A**). The PLS-DA and OPLS-DA plot showed a separation of the RT and COLD groups (**Supplementary Figures 3A, B**). A clear distinction was noted between COLD and RT plasma for negative ion modes presented in the permutation test plot (**Supplementary Figure 3C**). With the functional gene classifications in the KEGG database, the metabolome contents were organized by grouping metabolites into pathways. The KEGG pathway analysis showed that the metabolites were mainly enriched in lipid metabolism pathways and amino acid metabolism pathways (**Figure 6B**). The enrichment of plasma metabolites exists in glycerophospholipid metabolism, fatty acid biosynthesis, α-linolenic acid metabolism, biosynthesis of unsaturated fatty acids and linoleic acid metabolism suggested that lipid metabolism in porcine blood was active upon cold exposure (**Figure 6B**). Changes in plasma metabolites of pigs after cold exposure were presented in a clustered heat map (**Figure 6C**). In the heat map, the rows represented single metabolites and the columns represent the COLD or RT treated pigs. Significant decreased metabolites are displayed in green, and significant increased metabolites are displayed in red. The intensity of each color corresponds to the magnitude of the difference when compared with the average value. Supporting the combustion of stearic acid in SAT upon cold exposure, the circulating stearic acid content was significantly decreased (**Figure 6C**). We also found a reduction of α-linolenic acid in the plasma, a key PUFA promotes fatty acid remodeling and thermogenic activation (**Figure 6C**). Cold exposure markedly promoted the accumulation of organic acids (creatine, acamprosate, DL-3-phenyllactic acid, taurine) in plasma of pigs (**Figure 6C**). The heat map of Pearson correlation coefficients showed that the expression of heat generation related genes (RPS6KB2, CREB3L1, RPS6KB1, ATP5G1, NDUFV1, NDUFA10) in COLD SAT were positively associated with the contents of organic acids in COLD plasma (**Figure 6D**), but not COLD VAT (**Figure 6E**). Taken together, these results indicate that cold induced transcriptional changes in SAT and VAT might drive by a metabolic modulation of the circulating cold-adaptation machinery (**Figure 6F**).

## Discussion

It has been widely reported that cold exposure promotes the oxidize of fatty acids in brown adipocytes and the related beige adipocytes to generate heat through UCP1-dependent way in rodents and human (24). In our study, we investigated cold induced transcriptional and metabolic changes in subcutaneous and visceral adipose tissues, two fat depots with distinct metabolic phenotypes. And discovered the regulatory mechanisms in these two depots upon cold exposure in the absence of UCP1. Our results showed that cold exposure promotes the combustion of SFA accompanied by activating transcription process related to carboxylic acid metabolism in SAT. VAT exhibits active fatty acid metabolism with decreasing in several individual fatty acids upon cold exposure. Plasma metabonomic results indicated that cold induced absorption and consumption of stearic acid in SAT might drive by organic acids (such as creatine and taurine) for compensatory heat generation. Our results suggest that cold-induced change in adipose tissues of pigs is depot-specific and more investigations should be performed to uncover the regulatory mechanisms.

Cold exposure fuels combustion of triglyceride-derived fatty acids for heat-generating in adipose tissues (25). Our previous study showed that cold induced alterations in the composition and content of whole lipid profiles were considerable and with significant changes in the acyl-chain composition of TGs (26). Consistently, cold-treated UCP1 knock-in (KI) pigs also exhibited an extensive alteration in lipidomic profiles and significant reductions of total TG in inguinal white adipose tissue (iWAT). We also observed stable TG content in cold-treated porcine SAT. The content of TG was decreased in porcine VAT after cold treatment but without obvious decreased of adipocyte cell size, more experimental verification was needed for the change of TG content in VAT after cold treatment. Alteration of triglyceride storage leads to oxidative stress (27). We detected the markers of oxidative stress including TAOC, CAT, LDH and POD, which were assayed in the homogenates of the SAT and VAT. Though VAT exhibited a depletion of TG, the balance between oxidative and anti-oxidative was maintained in VAT upon cold exposure. The levels of oxidative- and antioxidative-associated substances (LDH, CAT, POD) were decreased in SAT after cold exposure. The decreased oxidative- and antioxidative level might related to inactive lipolysis in SAT upon cold exposure.

Fatty acid composition mildly changed upon cold exposure in both SAT and VAT. In particular, we found that cold exposure decreased stearic acid (C18:0) contents in SAT, while decreased lauric acid (C12:0), myristic acid (C14:0) and lignoceric acid (C24:0) contents in VAT. Moreover, the contents of SFA, MUFA and PUFA were not altered in VAT, while a significant SFA depletion was found in SAT. It was unclear if these fat-depots specific changes reflect utilization or export of these fatty acids. Previous studies shown that high-thermogenic adipose tissues, including BAT and cold-induced UCP1-positive WAT, exhibit cold-induced uptake of non-esterified fatty acids (28, 29). While in porcine backfat, one of so called UCP1-negative WAT, exhibits decreased SFA and MUFA content under cold acclimation (30). Our results showed that both SAT and VAT of pigs adapted to cold with a reduction in fatty acid content. It suggested that UCP1 might play an essential role in cold induced lipid and fatty acids metabolism in adipose tissues.

SAT and VAT have different adipogenic potential and metabolic characteristics has been known for decades (31). Our RNA-seq results showed that SAT exhibited an extensive alteration in transcriptional profiles with 1157 gene differentially expressed, 5 times as much as VAT with 289 DEGs upon cold exposure. GO enrichment analysis revealed enhanced biological processes (such as carboxylic acid biosynthetic process, organic acid biosynthetic process) and weakened immune response in SAT upon cold exposure, which might explain our finding that cold exposure diminishes the antioxidant capacity of porcine SAT mentioned above. While cold induced DEGs in VAT were enriched in adipocyte differentiation and immune response. These findings consistent with previous studies that cold-induced physiological change is heterogeneous in different fat depots of mice (32, 33). Besides, neither SAT nor VAT of cold-treated pigs exhibited significant up-regulation of lipid metabolism, fatty acid metabolism, lipid oxidation and fatty acid oxidation processes based on GO analysis, in conformity to previous reports on mice (34) and Tibetan piglets (35). These results demonstrated the commonality and diversity of SAT and VAT upon cold stress. Moreover, cold induced suppression of immune response in SAT and VAT of pigs was consist with a previous study in cold-treated mice (34), which indicated that downregulation of immune response in cold treated adipose tissues is independent of functional UCP1.

KEGG enrichment analysis of RNA-seq results of cold treat porcine SAT revealed that DEGs were abundant in pathways that facilitate regulating body temperature and recruitment of thermogenic capacity including PI3K-Akt, adipocytokine and apoptosis signaling pathway (36–38). KEGG enrichment analysis in VAT showed down-regulation of TGF1-β, PPAR, and FOXO signaling pathways upon cold exposure, indicating less effect of cold exposure on the browning (39)and adipogenesis (40) of VAT. According to the KEGG analysis, it seems that SAT of pigs takes more important part in heat generation than VAT. It has been revealed that cold exposure upregulates pathways related to the oxidative phosphorylation (OXPHOS) process and fatty acid β-oxidation to drive thermogenesis in SAT of mice (41, 42). We found that the genes related to the OXPHOS (COX5B, COX6B) were uniquely up-regulated in SAT and the genes related to fatty acids metabolism (ELOVL2, ELOVL4 and HADHA) uniquely up-regulated in VAT. Our data showed that ATGL, a key enzyme in response to cold-induced hydrolysis of intracellular TAG stores (43), was up-regulated in VAT after cold-treated. And UCP3, which contributes to the evolution of cold resistance in the Tibetan and Min pig (35), has higher expression in SAT after cold treat. Our results suggest that these two fat-depots might affect whole-body homeostasis upon cold exposure.

It is well known that cold exposure activates metabolic processes required to heat generation in plasma (44). KEGG enrichment analysis of our metabonomic results revealed that differential metabolites were abundant in lipid metabolism related processes including glycerophospholipid metabolism, fatty acid biosynthesis, α-linolenic acid metabolism, biosynthesis of unsaturated fatty acids and linoleic acid metabolism suggested that lipid metabolism in porcine blood was active upon cold exposure. According to a previous study in piglets, concentrations of FFA were decreased at 24 hours during cold exposure (45). In this study, we also observed a significant decrease in stearic acid, α-linolenic acid and eicosenoic acid in COLD plasma. Our recent study showed that cold exposure induced extensive increases in myristic acid (C14:0), palmitic acid (C16:0), linoleic acid (C18:2n-6c), eicosane acid (C20:1), α-linolenic acid (C18:3n-3) and γ-linolenic acid (C18:3n6) (18). Compared with the dynamic changes of SAT, VAT and plasma fatty acids in this study we mentioned above, it seemed that cold exposure promotes the absorb of myristic acid, palmitic acid and linolenic acid from VAT and Plasma to LDM. The content of stearic acid was specific diminished in SAT and plasma of pigs upon cold exposure. Stearic acid is a typical saturated fatty acid in the use as a fuel substrate upon cold exposure (46). A recent human trial declares that C18:0 intake causes mitochondrial fusion and increased fatty acid beta-oxidation *in vivo* (24). Consist with the up regulation of thermogenesis related genes in SAT, SAT selectively increase the absorb and use of SFAs during thermogenesis. In addition to activated lipid metabolism, overnight cold exposure accumulated high levels of carboxylic acids in pig plasma, including creatine, acamprosate, DL-3-phenyllactic acid and taurine, especially creatine and taurine, which promote heat generating in adipose tissues reportedly (47, 48). Connected with the upregulation of carboxylic acid biosynthetic process in SAT based on GO enrichment analysis of our RNA-seq results, we suggest that these carboxylic acids might activate compensatory thermoregulatory reaction which turn to saturated fatty acids as the main fuel in porcine reaction.

## Conclusions

In conclusion, our study investigated cold induced UCP1-independent effects of pig adipose tissues. We evaluated the similarity and difference in fatty acid profiles and transcriptome profiles of SAT and VAT in response to overnight cold exposure and performed plasma metabolome to explore the potential mechanisms. Future studies should be performed to verify our hypothesis and uncover the exact regulatory mechanism of the none UCP1 adipose tissues upon cold exposure to facilitate the development of new therapies for the treatment of epidemic obesity.

## Data Availability Statement

The datasets presented in this study can be found in online repositories. The names of the repository/repositories and accession number(s) can be found below: https://www.ncbi.nlm.nih.gov/, PRJNA779478.

## Ethics Statement

The animal study was reviewed and approved by ZJU20170466.

## Author Contributions

TS and ZX designed the experiments. YZ wrote the paper. YZ and ZX conducted the experiments. ZX, YZ, and LW analyzed the data. DL and QN provide regents and assisted interpretation. JX and XZ provide cold treated and collected samples. All authors have read and approved the final manuscript.

## Funding

The project was partially supported by the Zhejiang Provincial Key R&D Program of China (2021C02008), the Zaozhuang Talent Program Funding, and the “Hundred Talents Program” funding from Zhejiang University to TS.

## Conflict of Interest

Author JX and XZ are employed by Shandong Chunteng Food Co. Ltd.

The remaining authors declare that the research was conducted in the absence of any commercial or financial relationships that could be construed as a potential conflict of interest.

## Publisher’s Note

All claims expressed in this article are solely those of the authors and do not necessarily represent those of their affiliated organizations, or those of the publisher, the editors and the reviewers. Any product that may be evaluated in this article, or claim that may be made by its manufacturer, is not guaranteed or endorsed by the publisher.
