# Supplementary material for: Cold Exposure Induces Depot-Specific Alterations in Fatty Acid Composition and Transcriptional Profile in Adipose Tissues of Pigs
Source: Front Endocrinol (Lausanne). 2022 Feb 23;13:827523. doi: 10.3389/fendo.2022.827523 (PMC8905645; doi:10.3389/fendo.2022.827523)
Supplement: Supplementary file 1 [file DataSheet_1.docx]

***Supplementary Figures***

**
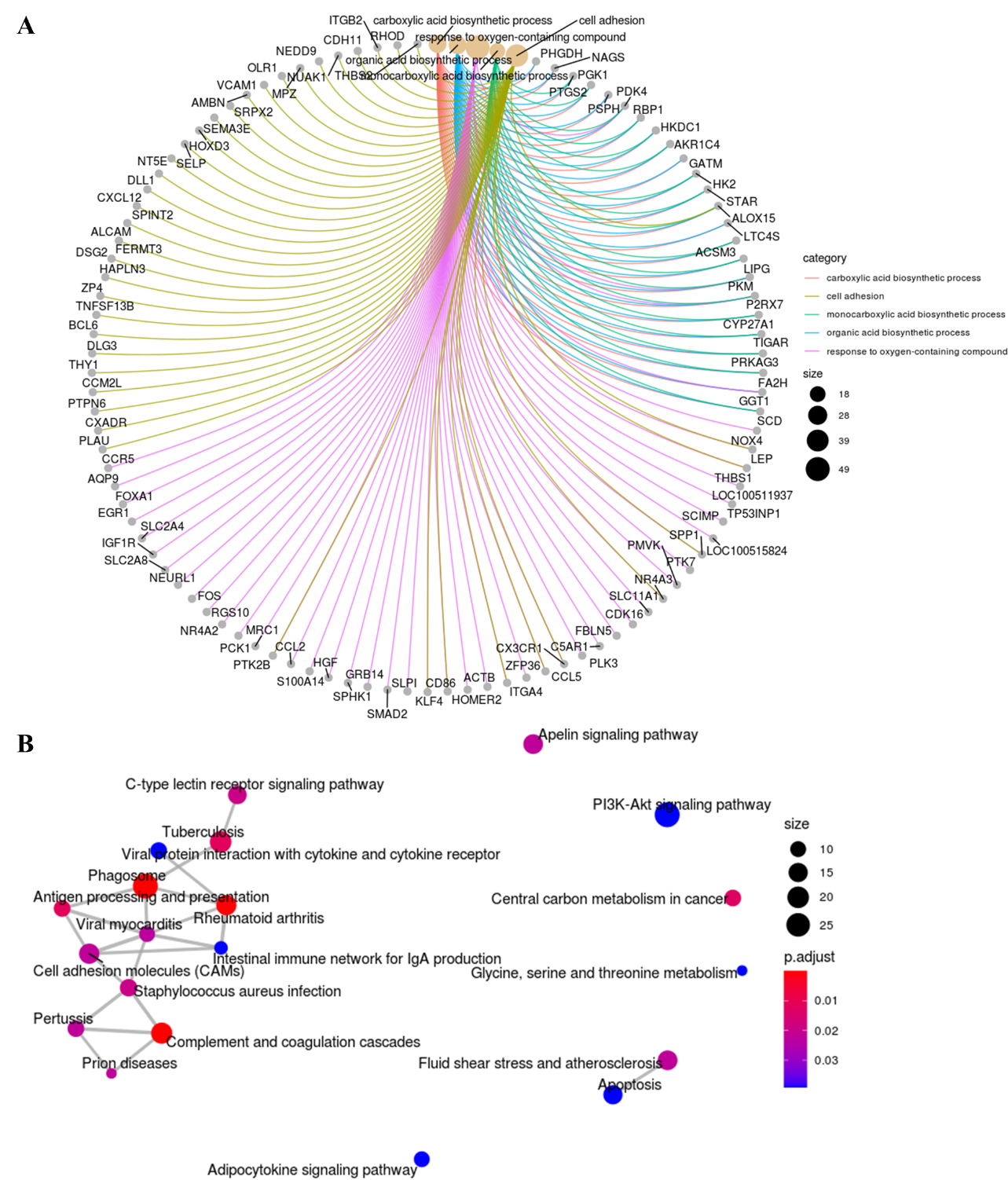
**

**Supplementary Figure 1.** Cold exposure induced transcription changes in porcine SAT. (A) The cnetplot depicts the linkages of the selected GO terms in the category of biological process. (B) The metaplot shows the linkages of pathways with common genes.

**
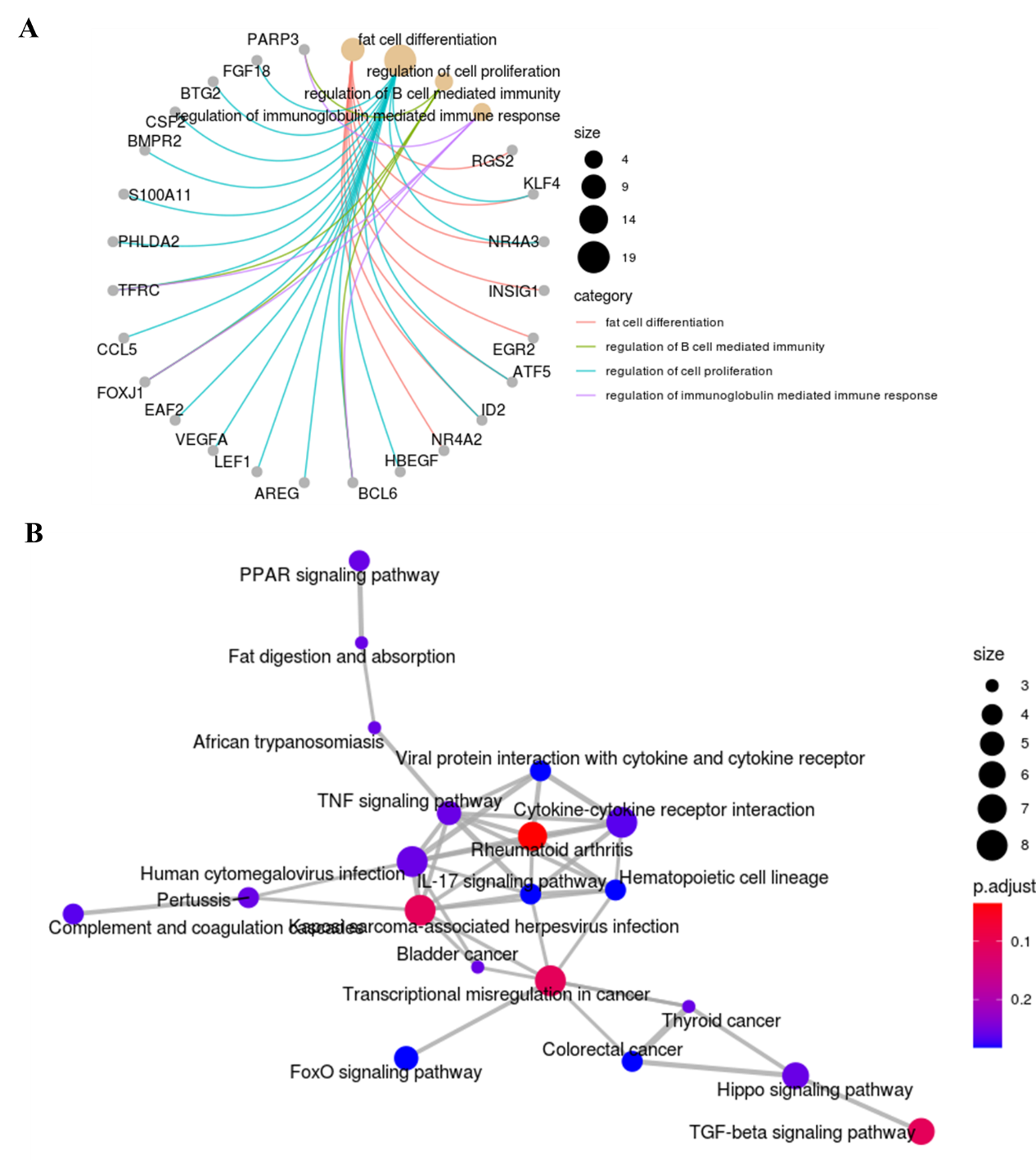
**

**Supplementary Figure 2.** Cold treated effects transcriptome profile in porcine VAT. (A) The cnetplot depicts the linkages of the selected GO terms in the category of BP. (B) The metaplot shows the linkages of pathways with common genes.


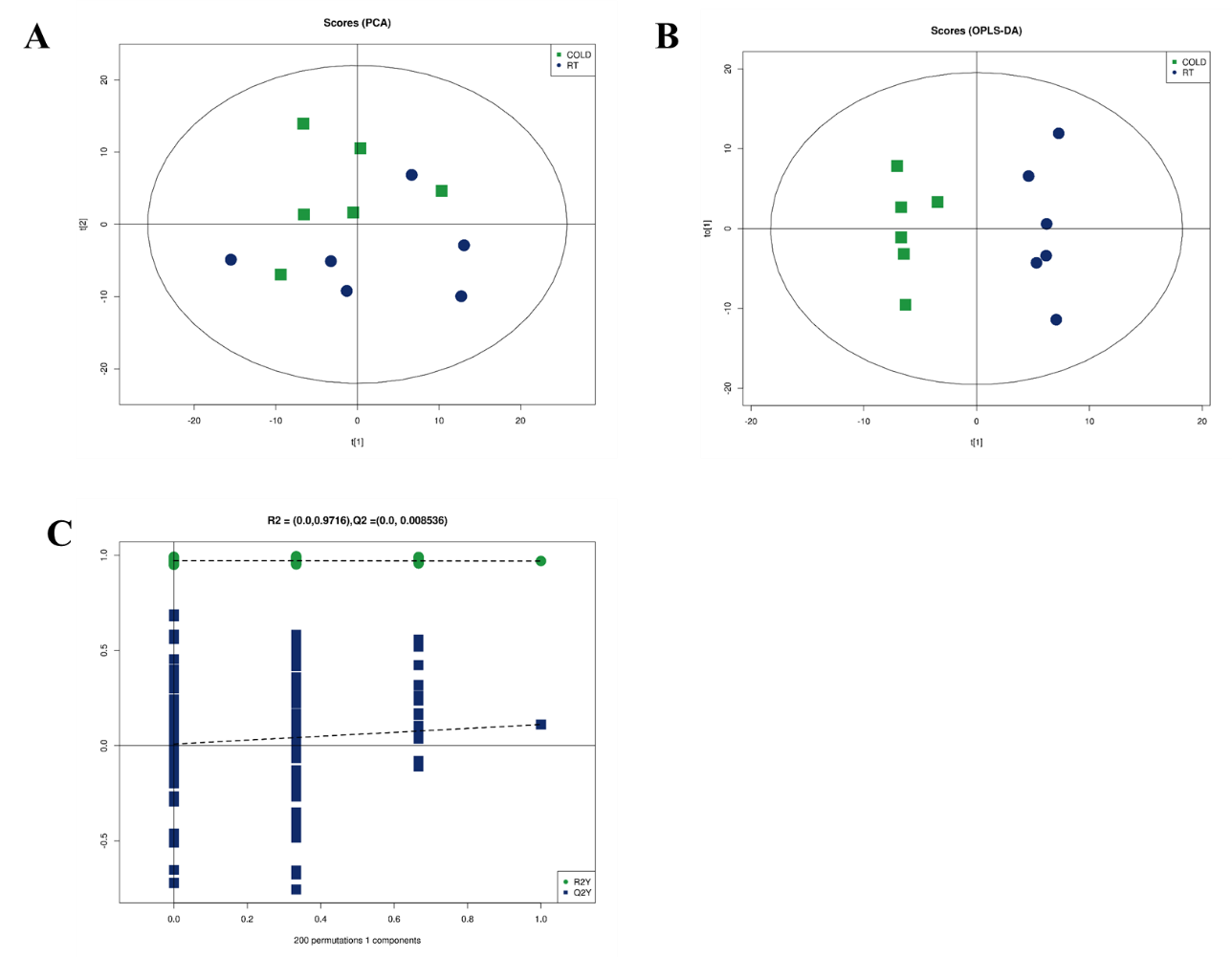


**Supplementary Figure 3.** Multivariate data analysis for LC-MS metabolite in control and cold-treated plasm. (A) Unsupervised principal component analysis (PCA) scores plot comparing RT with COLD samples in negative electrospray ionization mode (ESI−) metabolomics profiles of plasma. Blue and green symbols represent RT and COLD plasm samples, respectively. (B) Supervised OPLS-DA. Blue and green symbols represent RT and COLD samples, respectively. (C) Permutation test results of the OPLS-DA model in the negative ion mode.
