# Supplementary material for: Cold Exposure Induces Depot-Specific Alterations in Fatty Acid Composition and Transcriptional Profile in Adipose Tissues of Pigs
Source: Front Endocrinol (Lausanne). 2022 Feb 23;13:827523. doi: 10.3389/fendo.2022.827523 (PMC8905645; doi:10.3389/fendo.2022.827523)
Supplement: Supplementary file 2 [file DataSheet_2.docx]

***Supplementary Tables***

|  | **RT** |  | **COLD** |  |
| --- | --- | --- | --- | --- |
| **Items (g/100g)** | **Mean±SEM** |  | **Mean±SEM** | **P-value** |
| **C10:0** | **0.043±0.003** |  | **0.041±0.003** | **0.683** |
| **C12:0** | **0.053±0.002** |  | **0.047±0.003** | **0.170** |
| **C14:0** | **0.899±0.037** |  | **0.812±0.036** | **0.136** |
| **C15:0** | **0.029±0.003** |  | **0.031±0.004** | **0.720** |
| **C16:0** | **34.019±1.493** |  | **32.368±0.684** | **0.344** |
| **C17:0** | **0.187±0.013** |  | **0.191±0.023** | **0.871** |
| **C18:0** | **3.274±0.283** |  | **2.273±0.158** | **0.015** |
| **C20:0** | **0.191±0.016** |  | **0.170±0.011** | **0.310** |
| **C24:0** | **0.012±0.001** |  | **0.013±0.002** | **0.646** |
| **C14:1** | **0.006±0** |  | **0.004±0.001** | **0.051** |
| **C16:1** | **1.003±0.053** |  | **0.866±0.063** | **0.133** |
| **C18:1n9c** | **26.935±1.225** |  | **25.906±0.772** | **0.497** |
| **C20:1** | **0.555±0.04** |  | **0.492±0.026** | **0.228** |
| **C24:1** | **0.007±0** |  | **0.006±0** | **0.202** |
| **C18:2n6c** | **13.723±0.655** |  | **14.445±0.666** | **0.461** |
| **C18:3n6** | **0.025±0.002** |  | **0.024±0.002** | **0.881** |
| **C18:3n3** | **0.566±0.026** |  | **0.588±0.039** | **0.654** |
| **C20:2** | **0.594±0.036** |  | **0.609±0.008** | **0.694** |
| **C20:3n6** | **0.076±0.002** |  | **0.069±0.005** | **0.219** |
| **C20:3n3** | **0.083±0.005** |  | **0.084±0.005** | **0.800** |
| **C20:4n6** | **0.161±0.01** |  | **0.152±0.014** | **0.610** |
| **C22:2** | **0.009±0.001** |  | **0.008±0** | **0.396** |
| **C20:5n3** | **0.004±0** |  | **0.004±0** | **0.213** |
| **C22:6n3** | **0.008±0.002** |  | **0.007±0.001** | **0.760** |
| **SFA** | **38.706±4.017** |  | **35.946±1.632** | **0.192** |
| **MUFA** | **28.506±2.905** |  | **27.275±1.756** | **0.441** |
| **PUFA** | **15.248±1.614** |  | **15.991±1.624** | **0.489** |

**Table S1** Effects of cold exposure on fatty acid composition of SAT from finishing pigs.

Note: Statistical effect of cold exposure on fatty acid contents in SAT of pigs were analyzed by two-tailed Student’s t-test (n=5). SEM, standard of error means.

|  | **RT** |  | **COLD** |  |
| --- | --- | --- | --- | --- |
| **Items (g/100g)** | **Mean±SEM** |  | **Mean±SEM** | **P-value** |
| **C10:0** | **0.059±0.003** |  | **0.057±0.006** | **0.765** |
| **C12:0** | **0.067±0.003** |  | **0.057±0.005** | **0.147** |
| **C14:0** | **1.057±0.054** |  | **0.939±0.05** | **0.151** |
| **C15:0** | **0.026±0.002** |  | **0.020±0.002** | **0.144** |
| **C16:0** | **38.384±1.439** |  | **36.492±1.668** | **0.415** |
| **C17:0** | **0.168±0.013** |  | **0.148±0.018** | **0.401** |
| **C18:0** | **3.913±0.501** |  | **3.901±0.334** | **0.984** |
| **C20:0** | **0.200±0.013** |  | **0.192±0.007** | **0.598** |
| **C24:0** | **0.011±0.001** |  | **0.006±0.002** | **0.053** |
| **C14:1** | **0.006±0.001** |  | **0.005±0** | **0.184** |
| **C16:1** | **0.905±0.081** |  | **0.863±0.053** | **0.675** |
| **C18:1n9c** | **23.933±1.125** |  | **23.974±0.563** | **0.975** |
| **C20:1** | **0.443±0.024** |  | **0.411±0.027** | **0.386** |
| **C24:1** | **0.006±0.001** |  | **0.005±0.001** | **0.173** |
| **C18:2n6c** | **11.638±0.392** |  | **11.465±1.182** | **0.893** |
| **C18:3n6** | **0.025±0.001** |  | **0.020±0.002** | **0.110** |
| **C18:3n3** | **0.497±0.016** |  | **0.488±0.051** | **0.883** |
| **C20:2** | **0.428±0.022** |  | **0.418±0.037** | **0.826** |
| **C20:3n6** | **0.060±0.002** |  | **0.055±0.007** | **0.475** |
| **C20:3n3** | **0.064±0.005** |  | **0.072±0.007** | **0.347** |
| **C20:4n6** | **0.147±0.007** |  | **0.126±0.009** | **0.115** |
| **C22:2** | **0.007±0.001** |  | **0.007±0.001** | **0.542** |
| **C20:5n3** | **0.004±0** |  | **0.006±0.002** | **0.273** |
| **C22:6n3** | **0.005±0.001** |  | **0.008±0.001** | **0.195** |
| **SFA** | **43.880±4.091** |  | **41.814±4.434** | **0.466** |
| **MUFA** | **25.292±2.686** |  | **25.258±1.362** | **0.980** |
| **PUFA** | **12.874±0.952** |  | **12.660±2.883** | **0.881** |

**Table S2** Effects of cold exposure on fatty acid composition of VAT from finishing pigs

Note: Statistical effect of cold exposure on fatty acid contents in VAT of pigs were analyzed by two-tailed Student’s t-test (n=5). SEM, standard of error means.

**Table S3** Primers used for qPCR.

| Gene | Primer Name | Primer Sequence (5′-3′) |
| --- | --- | --- |
| Pig-Cox5b | Forward | CCAACCAGAACCAGACCAGGAAC |
|  | Reversed | GTACCATTCGCACACGGAGACG |
| Pig-Cox6b | Forward | CCAACCAGAACCAGACCAGGAAC |
|  | Reversed | GTACCATTCGCACACGGAGACG |
| Pig-Cox7a1 | Forward | GCTCTGGTCCGCTCCTTTA |
|  | Reversed | TCAGTTGCACCGCCCTTCG |
| Pig-Atp5j | Forward | CGCTTCCTGTCCGCAGAATCAC |
|  | Reversed | TGCCACCGCTGTAACACCAATG |
| Pig-Acsl | Forward | GTCGGATCAGAAGGTTGCCAGTG |
|  | Reversed | CTTGGGAAAGGATGGAGGGAATTGG |
| Pig-Acsl3 | Forward | CGTTTAAGCCCTGAACCGTGGAC |
|  | Reversed | GCTCAATGTCCGCCTGGTAATCTC |
| Pig-Elovl2 | Forward | AGGCTCTGGTCTCACCCTTTGG |
|  | Reversed | CTGGCTGTTATCACTCGGCTGAAG |
| Pig-Elovl4 | Forward | GTCTCCACTCCCTCCTCCCAAC |
|  | Reversed | AGGTCCAGCGGTAGAACTCCAC |
| Pig-Hadha | Forward | GTTCTTCCAGTGAGCGAGCCTTC |
|  | Reversed | GGTTCCTGTGATGAGTCCAGATGC |
| Pig-Hadhb | Forward | TTCCAACCAAGCCATGACCACAG |
|  | Reversed | TTAACTCTACACCACCTGCCACAAC |
| Pig-Hacd4 | Forward | GATGATTGTTGCCGTGTGCTGTG |
|  | Reversed | TTCCAGGCTAAGGGTCCACTCAG |
| Pig-Echs1 | Forward | ACCAGACCTTCCAGGACTGCTAC |
|  | Reversed | TCACACATCATAGCGAGTTCACAGC |
| Pig-Merc | Forward | AGCCCAGAGGAGCCAGAGTTAAG |
|  | Reversed | CAGAAGTGACAGGAGGGAGAGGAG |
| Pig-Hsl | Forward | ACCCTCGGCTGTCAACTTCTT |
|  | Reversed | ACTTTCTCCTCCTTGGTGCTAATCT |
| Pig-Atgl | Forward | GCACATCTCTCGAAGCACCA |
|  | Reversed | GCACATCTCTCGAAGCACCA |
| Pig-Ucp2 | Forward | ACCCAATGTCGCTCGTAATG |
|  | Reversed | GGAGGGCGTGAACCTGTC |
| Pig-Ucp3 | Forward | CGGGAGCAACAGGAAGTAC |
|  | Reversed | CCGAAGGCAGAGACAAAG |
| Pig-18s | Forward | CCCACGGAATCGAGAAAGAG |
|  | Reversed | TTGACGGAAGGGCACCA |
